# Altered Molecular Pathways in the Proteome of Cryopreserved Sperm in Testicular Cancer Patients before Treatment

**DOI:** 10.3390/ijms20030677

**Published:** 2019-02-05

**Authors:** Manesh Kumar Panner Selvam, Ashok Agarwal, Peter N. Pushparaj

**Affiliations:** 1 American Center for Reproductive Medicine, Cleveland Clinic, Cleveland, OH 44195, USA; manesh.balu@gmail.com; 2 Center of Excellence in Genomic Medicine Research, Jeddah 21589, Saudi Arabia; peter.n.pushparaj@gmail.com

**Keywords:** testicular cancer, sperm, unexplained infertility, cryopreservation, proteomics

## Abstract

Testicular cancer (TC) represents the most common cancer affecting men within the reproductive age and is often accompanied by major disturbances in semen parameters. Cryopreservation is recommended in these patients before initiating cancer treatment. Currently, there are no studies reporting the molecular mechanisms associated with altered semen quality in these men. The main objective of this study was to compare the sperm proteome of normozoospermic (motility >40%) and asthenozoospermic (motility <40%) TC patients with normozoospermic infertile men without cancer (control group). Pooled sperm samples from normozoospermic (*n* = 20), asthenozoospermic (*n* = 11) TC, and a control group (*n* = 9) were used for quantitative global proteomic profiling using liquid chromatography-tandem mass spectrometry. A total of 1085, 846, and 982 proteins were identified in normozoospermic TC, asthenozoospermic TC, and control groups, respectively. Functional analysis revealed mitochondrial dysfunction and altered cellular pathways in both normozoospermic and asthenozoospermic TC patients. Comparison of pathway analysis showed no significant difference in fertility-associated proteins/mechanism between the normozoospermic TC patients and infertile men. Western blot analysis revealed under-expression of NDUFS1 associated with mitochondrial dysfunction and overexpression of CD63 involved in sperm maturation in both normozoospermic and asthenozoospermic TC patients. Our proteomic results confirm that defective cellular pathways are associated with reproductive functions in both normozoospermic and asthenozoospermic TC patients before the start of cancer treatment.

## 1. Introduction

Testicular cancers (TCs) are diagnosed in men with an average reproductive age of 33 years. In the United States alone, 9310 new cases of TC were reported in 2018 and its incidence has been steadily increasing [1]. Infertile men possess increased risk of encountering TC compared to men from the general population [2,3,4,5]. Testicular cancer is one of the most curable cancers with a survival rate of 95% [6]. However, the majority of men with TC are diagnosed as infertile even before initiating cancer treatment [7,8,9,10,11]. Causes for infertility in these men include hormonal imbalance, intrinsic testicular damage, congenital abnormalities associated with testicular maturation, and spermatogenic failure [6,12].

The relationship between TC and impaired semen parameters before the onset of treatment is well established [13,14]. In general, a reduction in sperm concentration is seen in TC patients compared to other types of cancer [15,16]. In particular, asthenozoospermia (sperm motility < 40%) is prevalent in 30% to 45% of TC cases [15,17,18]. Several studies have reported sub-normal semen parameters and poor fertilization rates in men with TC [11,19,20,21,22,23]. However, 50% of TC patients are found to have normal semen parameters (normozoospermia) according to WHO 2010 reference values [6,13]. The reason for infertility in these men with normal semen parameters is unclear.

Unexplained male infertility is a multifactorial disorder and conventional semen analysis fails to explain the etiology of infertility in such cases [24]. Furthermore, asthenozoospermia is a common cause of male infertility and it is observed in 18% of infertile men [25] and accounts for up to 81% of all abnormal semen analyses [26]. Over the past decade, the proteomic platform has been used to study the changes in the protein profiles of spermatozoa [27,28,29,30,31,32]. Advancement in sperm proteomic research has provided the molecular basis of unexplained infertility [31]. Several sperm proteomic studies on normozoospermic infertile men have reported changes at the subcellular level of spermatozoa [32,33,34,35]. These changes are associated with proteins related to sperm capacitation and acrosome reaction that are essential for the fertilization of oocyte [26,36].

The majority of TC patients are referred for sperm banking by their oncologists before the start of their cancer treatment. Fertility history in the majority of these patients is not established. In addition, they are not referred to a fertility specialist because of the urgency of oncological treatment. Therefore, to understand the possible underlying cause(s) of infertility in TC patients, we compared the sperm proteome of normozoospermic and asthenozoospermic TC patients with that of normozoospermic infertile men without cancer (control group).

## 2. Results

### 2.1. Semen Parameters

Semen parameters of the normozoospermic and asthenozoospermic TC patients, and control group are presented in Supplementary Materials Table S1. No difference was seen in the sperm concentration between normozoospermic TC patients (*n* = 20) and normozoospermic infertile men without cancer (control group) (*n* = 20), and between asthenozoospermic TC patients (*n* = 20) and the control group. Sperm motility was significantly (*p* < 0.0001) decreased in asthenozoospermic TC patients (Supplementary Materials Table S1).

### 2.2. Sperm Proteome of TC Patients and Normozoospermic Infertile Men

**Figure 1:**
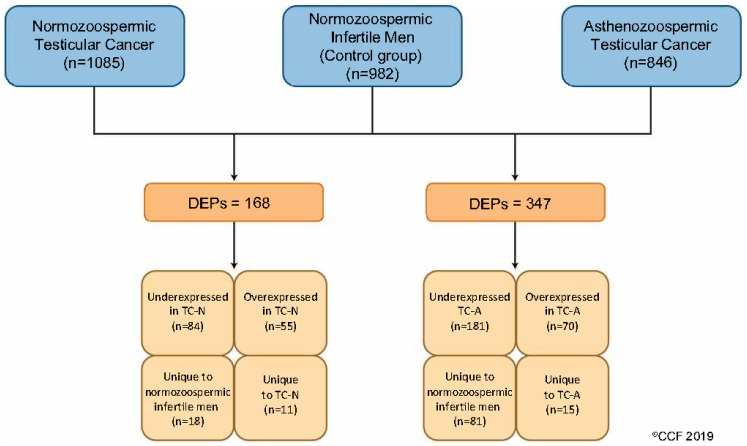
Differentially expressed sperm proteins (DEPs) in normozoospermic and asthenozoospermic testicular patients and normozoospermic infertile men without cancer (control group). TC-N: testicular cancer normozoospermic, TC-A: testicular cancer asthenozoospermic.

Liquid chromatography-tandem mass spectrometry (LC-MS/MS) detected a total of 1085, 846, and 982 proteins in normozoospermic TC, asthenozoospermic TC and control groups, respectively. Based on the normalized spectral abundance factor (NSAF) ratio and protein abundance, 168 differentially expressed proteins (DEPs) were identified in normozoospermic TC and 347 in asthenozoospermic TC patients compared with the control group. The overexpressed and under-expressed DEPs, and unique proteins are shown in Figure 1.

### 2.3. Biological Pathways Dysregulated in Spermatozoa of Normozoospermic and Asthenozoospermic TC Patients

Ingenuity pathway analysis (IPA) revealed phagosome maturation, sirtuin signaling pathway, mitochondrial dysfunction, atherosclerosis signaling, and remodeling of epithelial adherens junctions as the top five canonical pathways in normozoospermic TC patients (Table 1). Mitochondrial dysfunction, oxidative phosphorylation, sirtuin signaling pathway, protein ubiquitination pathway, and phagosome maturation were identified as top canonical pathways in asthenozoosp ermic TC patients (Table 1). 

**Figure 2:**
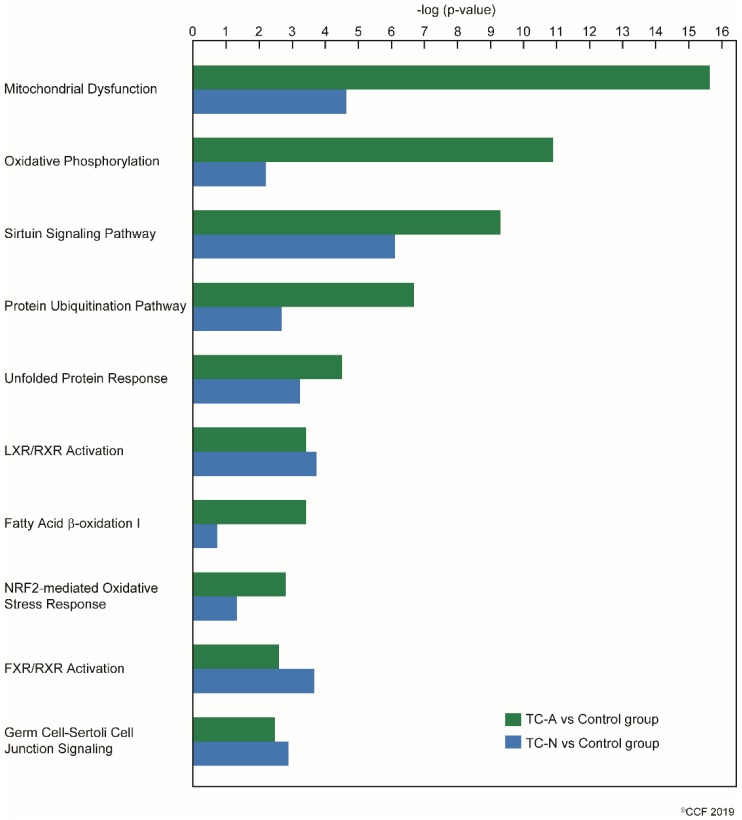
A comparison pathway analysis depicting the canonical pathways associated with reproductive process function between the TC patients with normal and abnormal semen parameters when compared with normozoospermic infertile men (control group). TC-N: testicular cancer normozoospermic, TC-A: testicular cancer asthenozoospermic.

**Figure 3:**
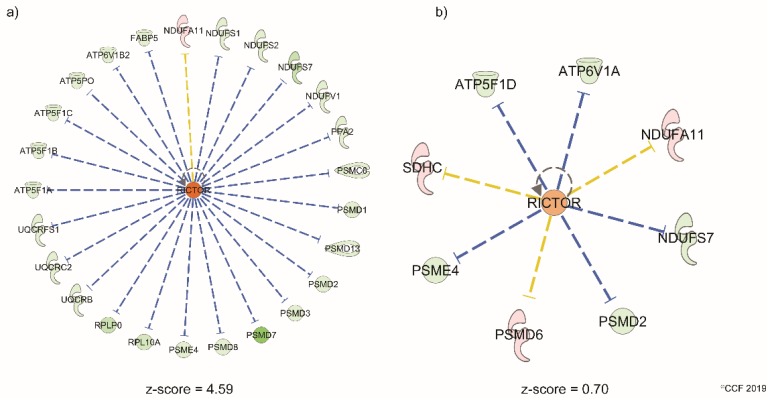
Differentially expressed proteins under the regulation of RICTOR in (**a**) asthenozoospermic testicular cancer group, (**b**) normozoospermic testicular cancer group. A z-score >2 and <−2 is considered significant. Dashed lines: indirect interaction, blue color: leads to inhibition, yellow color: findings inconsistent with state of downstream molecule.

Canonical pathways-related reproductive function and their significance in both normozoospermic and asthenozoospermic TC patients are shown in Figure 2. Upstream regulator analysis revealed rapamycin-insensitive companion of mammalian target of rapamycin (RICTOR) function was significantly activated in asthenozoospermic TC patients with a z-score of 4.59. No significant activation of RICTOR was seen in normozoospermic TC patients. Differentially expressed sperm proteins associated with RICTOR are shown in Figure 3.

Functional analysis of DEPs identified the top pathways associated with disease and disorders, molecular and cellular functions in both normozoospermic and asthenozoospermic TC groups. Inflammatory response was identified as the top pathway in disease and disorders, and cellular compromise in molecular and cellular functions. However, the pathways associated with physiological system development and function were identified only in the asthenozoospermic TC patients (Table 2). Forty DEPs were involved in reproductive system development pathway (Table 2).

### 2.4. Protein Networks and Biofunctions Affected in Asthenozoospermic TC Group

**Figure 4:**
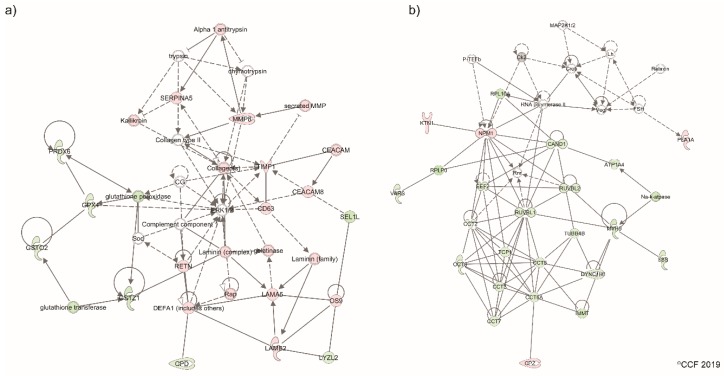
Network showing differentially expressed proteins associated with (**a**) cellular assembly and organization, cell-to-cell signaling and interaction, and reproductive system development pathways, (**b**) cellular compromise, inflammatory response, infectious diseases in asthenozoospermic testicular cancer group. Dashed lines: indirect interaction, continuous lines: direct interaction.

Bioinformatic analysis identified the DEPs associated with sperm function and fertilization process that were altered in the asthenozoospermic TC patients (Table 3). Network analysis revealed the involvement of sperm proteins in cellular assembly and organization, cell-to-cell signaling and interaction, reproductive system development and function (Figure 4a), and cellular compromise, inflammatory response, and infectious diseases (Figure 4b).

### 2.5. Western Blot Analysis of Validated DEPs

**Figure 5:**
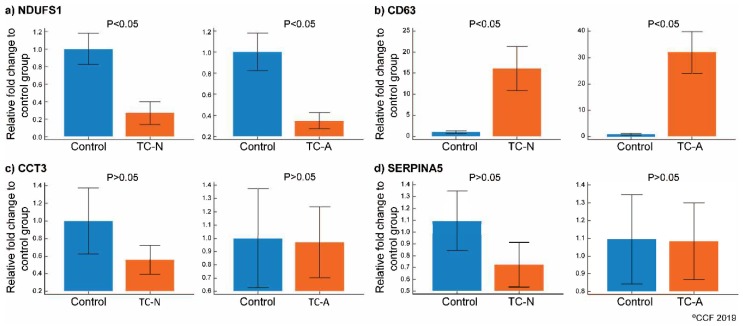
Protein expression levels of the differentially expressed proteins (DEPs) selected for validation by Western blot in normozoospermic (*n* = 10), asthenozoospermic (*n* = 10) TC patients with control group (*n* = 7). (**a**) NDUFS1, (**b**) CD63, (**c**) CCT3, (**d**) SERPINA5. Control: normozoospermic infertile men without cancer, TC-N: testicular cancer normozoospermic, TC-A: testicular cancer asthenozoospermic.

Of the four validated proteins CD63 antigen (CD63) was overexpressed and NADH:ubiquinone oxidoreductase core subunit S1 (NDUFS1) was under-expressed (*p* < 0.05) in both normozoospermic and asthenozoospermic TC groups (Figure 5a,b). Chaperonin containing TCP1 subunit 3 (CCT3) and plasma serine protease inhibitor (SERPINA5) expression was comparable in the three groups (Figure 5c,d).

## 3. Discussion

Testicular cancer-associated male infertility is due to the side effect of aggressive oncology treatment [37]. Treatment options for TC such as radiation- and chemotherapy damages the gonads and results in impaired spermatogenesis process [12,38,39]. To improve the quality of life, fertility preservation is recommended in TC patients [40]. Sperm cryopreservation before treatment is a cost-effective strategy to establish a successful pregnancy [41]. Therefore, it is crucial to analyze and define the patient’s pretreatment fertility and improve our understanding of the impact of TC and future fertilization potential in these men. Several studies have reported the successful use of cryopreserved sperm of TC patients for fathering a child [42,43,44]. Záková et al. [17] reported a pregnancy rate of 34.8% after using the cryopreserved sperm from TC patients. Similarly, conception rate was 30.4% in the men before diagnosis of TC [22]. Poor semen quality may be the possible reason for low pregnancy rate in these men. 

In TC patients, asthenozoospermia is well documented [11,45]. However, certain populations of TC patients also have normal semen parameters before treatment [46] and their fertility status remains questionable. In the current study, semen analysis results showed no significant difference in the sperm concentration and motility of normozoospermic TC patients prior to cancer treatment compared with the control group. Hence, it is important to understand the changes in the molecular mechanisms associated with sperm function in normozoospermic TC men utilizing the proteomic approach.

The sperm proteome is highly complex and requires high throughput instruments such as LC-MS/MS to detect the maximum number of peptides and proteins [47,48,49,50,51]. In the current experiment, we also used LC-MS/MS to profile sperm proteins in TC patients and the control group. Until now, the majority of the sperm proteomic studies have been carried out on asthenozoospermic [52,53,54,55,56] or normozoospermic infertile men [32,33,34,35]. However, no reports are available on the proteomic changes associated with spermatozoa in normozoospermic and asthenozoospermic TC patients. Hence, comparing the proteome profiles of normozoospermic and asthenozoospermic TC patients with normozoospermic infertile men (control group) may provide an insight into the subcellular changes responsible for male infertility in these patients before initiating cancer treatment. Our proteomic results revealed fewer number of DEPs in spermatozoa of asthenozoospermic TC patients and the majority of the identified DEPs were either under-expressed or absent. This finding suggests that certain biological pathways are dysregulated in the spermatozoa, thus affecting sperm cell homeostasis in asthenozoospermic TC patients.

Progression of TC impairs the spermatogenesis process [12]. Any defects during the sperm formation may also contribute to mitochondrial dysfunction. Mitochondrion is the power house of sperm and its proper functioning is crucial for motility, hyperactivation, capacitation, acrosome reaction, and fertilization of spermatozoa [57]. Bracke et al. [26] and Cao et al. [58] reviewed sperm proteomic studies and reported that energy metabolism was dysfunctional in asthenozoospermic men. In asthenozoospermic TC patients in the current study, we noted that mitochondrial dysfunction was the top canonical pathway affected followed by oxidative phosphorylation (Table 1). Even though the dysfunctional mitochondrial pathway was also noted to be affected in the normozoospermic TC patients, the dysregulation was comparatively more pronounced in the asthenozoospermic TC patients (Figure 2). Validation of the under-expressed mitochondrial protein NDUFS1 using Western blot supports our proteomic findings. This inner mitochondrial membrane protein is involved in the transfer of electrons in the oxidative phosphorylation process. Under-expression of NDUFS1 suggests of mitochondrial dysfunction. In addition, NDUFS1 is under the regulation of the RICTOR signaling pathway which regulates the spermatogenesis process and helps in the maintenance of the blood–testis barrier [59,60]. Upstream regulator analysis predicted the activation state of RICTOR in asthenozoospermic TC patients (Figure 3). Hence, activation of RICTOR and under-expression of NDUFS1 implicates spermatogenic failure and mitochondrial dysfunction in asthenozoospermic TC patients. However, under-expression of NDUFS1 without activation of RICTOR is suggestive of mitochondrial dysfunction without compromising sperm motility in normozoospermic TC patients.

The functionality of spermatozoa is regulated by the molecular pathways associated with cellular functions. Our bioinformatic analysis revealed that the cellular compromised pathway was affected in both normozoospermic and asthenozoospermic TC patients (Table 2). Siva et al. reported [61] the pathways compromising sperm functions such as stress response and sperm maturity were dysregulated in asthenozoospermic males. Proteins involved in vesicular trafficking were also found to be deregulated in asthenozoospermic men [52]. Our proteomic results identified proteins interacting with each other in the cellular compromise network (Figure 4b) were altered in asthenozoospermic TC patients. Validation of CD63 protein identified in the network further strengthens our bioinformatic results. Furthermore, CD63 is an exosomal marker protein [62] and is involved in the cellular molecule trafficking [63]. In general, spermatozoa fuse with the epididymosomes (exosomes) during its epididymal transit and undergoes maturation [64]. Aberrant expression of CD63 may affect the vesicle fusion and result in the production of immature spermatozoa. In the present study, using the Western blot technique we demonstrated the overexpression of CD63 in normozoospermic as well as asthenozoospermic TC patients (Figure 5). This finding suggests that the sperm maturation process is defective in TC patients.

A deep insight into the proteins related to the fertilization process provides a better understanding about the fertilization potential of spermatozoa. We identified the proteins involved in the reproductive system and developmental functions that were dysregulated in asthenozoospermic TC patients. Chaperonin protein CCT3 involved in sperm–egg and sperm–zona pellucida binding was detected in the network (Figure 4b) [65]. Under-expression of CCT3 in asthenozoospermic TC patients indicates a compromised fertilization process. Serpin family protein SERPINA5 is implicated in the fertilization process, as it inhibits the binding and penetration of sperm [66] and plays a prominent role in male infertility [67]. Our proteomic results showed overexpression of SERPINA5 in asthenozoospermic TC patients (Table 3). However, Western blot validation of CCT3 and SERPINA5 did not show significant difference in the expression pattern among TC patients and control group. These discrepancies may be due to the reduced specificity and sensitivity of the conventional Western blot technique compared to robust LC-MS/MS technique. Our proteomic findings suggest that even though normozoospermic TC patients exhibit normal semen parameters, sperm proteins associated with the fertilization process are dysregulated in these men.

To our knowledge, this is the first proteomic study to investigate the molecular pathways associated with altered reproductive functions in normozoospermic and asthenozoospermic TC patients. A limitation of the current study was that we did not have a follow-up of the fertility status of TC patients. We have demonstrated by use of proteomic analysis that mitochondrial dysfunction is the main cause of infertility in TC patients. Our data suggests that NDUFS1 and CD63 may serve as potential protein biomarkers for mitochondrial dysfunction and sperm maturation in TC patients. Further research on fertility associated proteins CCT3 and SERPINA5 is warranted to establish their utility as clinical biomarkers in these men.

## 4. Materials and Methods

### 4.1. Study Participants

This study was approved by the Institutional Review Board (IRB) of Cleveland Clinic. All the participants signed an informed written consent at the time of sperm banking at the Andrology Center, Cleveland Clinic. The cryopreserved semen samples from TC patients before starting cancer therapy were used for proteomic analysis in compliance with the Minimum Information about a Proteomics Experiment (MIAPE) guidelines of the Human Proteome Organization’s Proteomics Standards Initiative (HUPO-PSI) for reporting proteomics studies [68]. Patients also consented to the use of discarded samples in research.

The inclusion criteria was the use of cryopreserved samples from all patients before the start of any cancer therapy. This was regardless of the stage or specific disease subtype. Based on the WHO 2010 guidelines, semen samples were divided into normozoospermic (motility > 40%), *n* = 20; and asthenozoospermic (motility < 40%), *n* = 20. We also included a control group (*n* = 20) comprising of normozoospermic infertile men without cancer. These infertile men had not fathered a child in the past 2 years before their enrollment in the study. Female partners of these infertile men were reported to have normal reproductive health following general gynecological evaluation.

### 4.2. Semen Analysis and Cryopreservation

Semen samples were collected after 2–3 days of sexual abstinence and allowed to liquefy completely for 20–30 min at 37 °C. Semen volume, and sperm motility and concentration were evaluated according to the WHO 2010 guidelines [69]. Semen samples from TC patients and control group were cryopreserved in TEST-yolk buffer (TYB; Irvine Scientific, Santa Ana, CA, USA) using the slow-freezing protocol [70].

### 4.3. Sperm Protein Extraction and Quantification

Cryopreserved samples were thawed at 37 °C for 20 min and centrifuged at 4000× *g* for 10 min to isolate spermatozoa. The sperm pellet was washed four times with phosphate buffered saline (PBS; Irvine Scientific, Santa Ana, CA, USA) and centrifuged at 4000× *g* for 10 min at 4 °C. Radio-immunoprecipitation assay (RIPA; Sigma–Aldrich, St. Louis, MO, USA) buffer supplemented with Protease Inhibitor Cocktail, cOmpleteTM ULTRA Tablets, EDTA-free (Roche, Mannheim, Germany) was added to sperm pellet (100 µL RIPA/10^6^ sperm) and left overnight at 4 °C for cell lysis. Samples were centrifuged at 10,000× *g* for 30 min at 4 °C and the supernatant was transferred to a new centrifuge tube. Protein quantification in the fractions was performed using the Pierce BCA Protein Assay kit (Thermo Fisher Scientific, Waltham, MA, USA) according to the manufacturer’s instructions.

### 4.4. Liquid Chromatography-Tandem Mass Spectrometry

Pooled samples from 20 normozoospermic men with TC, 11 from asthenozoospermia men with TC and 9 from control group were used for global proteomic analysis by LC-MS/MS. The samples in each pool were mixed with SDS Page buffer and separated on a 1D gel and run in triplicates. For the protein digestion step, the bands were cut to minimize excess polyacrylamide, and divided into a number of smaller pieces. The gel pieces were washed with water and dehydrated in acetonitrile. The bands were then reduced with dithiothreitol and alkylated with iodoacetamide. Subsequently all bands were digested in-gel using trypsin, by adding five μL of 10 ng/μL trypsin in 50 mM ammonium bicarbonate and incubating overnight at room temperature to achieve complete digestion. The peptides formed were extracted from the polyacrylamide in two aliquots of 30 μL 50% acetonitrile with 5% formic acid. These extracts were combined and evaporated to <10 μL in the Speedvac and then resuspended in 1% acetic acid to make up a final volume of ~30 μL for LC-MS analysis.

The LC-MS system was a Finnigan LTQ-Orbitrap Elite hybrid mass spectrometer system. The HPLC was performed using a Dionex 15 cm *×* 75 μm id Acclaim Pepmap C18, 2 μm, 100 Å reversed phase capillary chromatography column as described in our previous publication [71]. The data was analyzed using all CID spectra collected in the experiment to search the human reference sequence databases (http://www.hprd.org/) with the search program Mascot and Sequest. These search results were then uploaded into the program Scaffold (Proteome Software Inc., Portland, OR, USA; version 4.0.6.1). The abundance of each protein in the pool was classified as very low, low, medium or high based on the number of spectral counts. The NSAF ratio was calculated to categorize the expression profile of DEPs as under-expressed, overexpressed or unique to one of the groups [71].

### 4.5. Bioinformatic Analysis

Functional pathway analysis of the DEPs was done using the IPA software (Qiagen, Hilden, Germany). The IPA program facilitates the evaluation of top canonical pathways, diseases, and bio-functions and upstream regulators related to the DEPs. Comparison analysis was also carried out between the two analyzed datasets (normozoospermic TC vs. control group and asthenozoospermic TC vs. control group) to identify the differences in the canonical pathways regulated by the DEPs.

### 4.6. Protein Selection and Validation by Western Blot

To validate the global proteomic results, sperm proteins related to reproductive function were selected for validation by Western blot (WB). This was performed in a different set of samples from normozoospermic and asthenozoospermic TC patients and control group to maintain the biological variability. The criteria for the selection of DEPs for validation by WB included: (1) proteins involved in reproductive system development and function; (2) proteins involved in the top canonical pathways; and (3) proteins with a well-described function in the literature. Four proteins (CCT3; CD63, NDUSF1, and SERPINA5) were selected for validation by WB in individual samples from normozoospermic TC (*n* = 10), asthenozoospermic TC (*n* = 10), and control (*n* = 7) groups. A total of 20 µg of protein per sample was loaded into a 4%–15% SDS–PAGE for 2 h at 90 V. The resolved protein bands were then transferred onto polyvinylidene difluoride (PVDF) membranes and for each protein analysis, specific primary antibodies were incubated at 4 °C overnight (Supplementary Table S2). The membranes were incubated with the secondary antibody at room temperature for 1 h and finally reacted with enhanced chemiluminescence (ECL) reagent (GE Healthcare, Marlborough, MA, USA) for 5 min. Membranes were exposed to Chemi-Doc (ChemiDoc™ MP Imaging System, Bio-Rad, Hercules, CA, USA) to detect the chemiluminescence signals.

All the PVDF membranes used for protein identification were subjected to total protein staining. The membranes were briefly washed twice for 10 min in distilled water and stained with colloidal gold total protein stain (Bio-Rad, Hercules, CA, USA) for 2 h at room temperature by gentle shaking. Stained membranes were washed twice with distilled water for 10 min, and the densitometry image was captured using the colorimetric mode on Chemi-Doc (ChemiDoc™ MP Imaging System, Bio-Rad).

### 4.7. Statistical Analysis

Data analysis was performed using MedCalc Statistical Software (version 17.8; MedCalc Software, Ostend, Belgium). After testing for normal distribution using the Kolmogorov–Smirnov test, the Mann–Whitney test was carried out to compare the semen parameters of the normozoospermic and asthenozoospermia TC patients with that of control group, and a *p* < 0.05 was considered as significant. The same test was used to compare the expression levels of the proteins validated using western blot technique in both the groups.

## Figures and Tables

**Table 1:** List of differentially expressed proteins involved in top 5 canonical pathways associated with normozoospermic and asthenozoospermic testicular cancer patients.

Groups	Canonical Pathways	−log (*p*-Value)	DEPs
**Normozoospermic** **TC patients**	Phagosome Maturation	7.19	DYNC1H1,M6PR,TUBB3,MPO,PRDX1,TUBB4B,TUBA3C/TUBA3D,CANX,ATP6V1A,NAPA
	Sirtuin Signaling Pathway	6.15	SLC25A6,PPIF,ATP5F1D,NDUFS7,NDUFA11,CPT1B,TUBA3C/TUBA3D,HIST1H1D,SDHC,SLC25A5,VDAC3,VDAC1
	Mitochondrial Dysfunction	4.67	ATP5F1D,NDUFA11,NDUFS7,CPT1B,SDHC,OGDH,VDAC1,VDAC3
	Atherosclerosis Signaling	4.61	ALB,APOB,APOA4,LPL,SERPINA1,COL18A1,CLU
	Remodeling of Epithelial Adherens Junctions	3.99	TUBB3,TUBB4B,TUBA3C/TUBA3D,ACTN4,ACTN1
**Asthenozoospermic** **TC patients**	Mitochondrial Dysfunction	15.7	HSD17B10,NDUFV1,NDUFS7,ATP5F1A,ATP5PO,ATP5S,VDAC3,UQCRB,VDAC2,PDHA1,ATP5F1C,MTND5,NDUFS1,ATP5F1B,NDUFA11,UQCRC2,NDUFS2,UQCRFS1,GPX4,VDAC1,OGDH
	Oxidative Phosphorylation	10.9	NDUFV1,ATP5F1C,MTND5,NDUFS1,ATP5F1B,NDUFS7,NDUFA11,ATP5F1A,UQCRC2,ATP5PO,NDUFS2,UQCRFS1,ATP5S,UQCRB
	Sirtuin Signaling Pathway	9.31	NDUFV1,PPIF,NDUFS7,ATP5F1A,VDAC3,VDAC2,PDHA1,ATP5F1C,NDUFS1,MTND5,ATP5F1B,NDUFA11,UQCRC2,TUBA3C/TUBA3D,NDUFS2,UQCRFS1,VDAC1,SLC25A5,LDHA
	Protein Ubiquitination Pathway	6.67	PSMD7,PSMD13,HSPH1,HSPA9,TRAP1,PSMD3,PSMD8,UCHL3,USP7,PSMC6,PSMD2,DNAJB11,PSMD1,DNAJB1,HSPA4L
	Phagosome Maturation	5.3	DYNC1H1,CTSD,MPO,TUBB4B,CTSB,TUBA3C/TUBA3D,CANX,PRDX6,ATP6V1B2,HLA-DRB5

**Table 2:** List of pathways associated with diseases and disorders, molecular and cellular functions, physiological system development and functions in normozoospermic and asthenozoospermic testicular cancer patients.

Groups	Normozoospermic TC			Asthenozoospermic TC		
Category	Pathways	*p*-Value	Number of DEPs	Pathways	*p*-Value	Number of DEPs
**Disease and disorders**	Inflammatory response	2.36 × 10^−14^–6.15 × 10^−7^	46	Inflammatory Response	1.96 × 10^−22^–2.37 × 10^−3^	84
	Cancer	4.65 × 10^−11^–7.06 × 10^−6^	142	Cancer	3.9 × 10^−11^–2.19 × 10^−3^	228
	Organism injury and abnormalities	4.65 × 10^−11^–7.06 × 10^−6^	142	Organism injury and abnormalities	3.9 × 10^−11^–2.39 × 10^−3^	232
	Reproductive system disease	1.3 × 10^−10^–7.06 × 10^−6^	104	Metabolic disease	1.2 × 10^−10^–2.36 × 10^−3^	90
	Neurological disease	3.21 × 10^−10^–7.06 × 10^−6^	47	Gastrointestinal disease	2.02 × 10^−9^–2.13 × 10^−3^	214
**Molecular and cellular functions**	Cellular compromise	2.36 × 10^−14^–2.45 × 10^−7^	30	Cellular compromise	1.96 × 10^−22^–1.88 × 10^−3^	59
	Protein synthesis	1.06 × 10^−11^–2.54 × 10^−6^	39	Protein synthesis	3.16 × 10^−14^–2.36 × 10^−3^	74
	Post-translational modification	2.17 × 10^−11^–6.41 × 10^−10^	18	Protein degradation	3.93 × 10^−14^–2.36 × 10^−3^	39
	Protein degradation	2.17 × 10^−11^–6.41 × 10^−10^	26	Cellular assembly and organization	8.2 × 10^−11^–2.36 × 10^−3^	46
	Lipid metabolism	4.3 × 10^−11^–6.79 × 10^−6^	26	Post-translational modification	2.08 × 10^−10^–2.36 × 10^−3^	52
**Physiological system development and functions**	NA	NA	NA	Reproductive system development and function	4.14 × 10^−10^–2.05 × 10^−3^	40
	NA	NA	NA	Hematological system development and function	3.85 × 10^−7^–2.37 × 10^−3^	38
	NA	NA	NA	Immune cell trafficking	3.85 × 10^−7^–2.37 × 10^−3^	39
	NA	NA	NA	Organ development	9.75 × 10^−6^–2.4 × 10^−3^	36
	NA	NA	NA	Cardiovascular system development and function	2.62 × 10^−5^–2.23 × 10^−3^	40

**Table 3:** Reproductive system development and functions affected due to altered expression of DEPs in asthenozoospermic testicular cancer patients.

Function	*p*-Value	DEPs
Binding of sperm	4.14 × 10^−10^	CCT2,CCT3,CCT5,CCT6A,CCT7,CCT8,PRSS37,SPAM1,TCP1,VDAC2
Spermatogenesis	0.000429	APOB,ATP1A4,GPX4,H2AFX,HSD17B4,HSPA4L,KIAA1324,NPEPPS,NUP210L,PRSS37
Maturation of sperm	0.00114	CLU,TPP2
Function of sperm	9.75 × 10^−6^	AKAP4,ATP1A4,ATP2B4,PRSS37,SPESP1,VDAC1
Cell movement of sperm	0.000062	AKAP4,APOB,ATP1A4,ATP2B4,CHDH,GAPDHS,SPAG6,VDAC3
Development of genital organ	0.00205	ALB,APOB,ATP1A4,GPX4,H2AFX,HSD17B4,HSPA4L,KIAA1324,NPEPPS,NUP210L
Fertilization	0.000137	AKAP3,AKAP4,APOB,ATP1A4,PRSS37,SERPINA5,SPAM1,SPESP1,VDAC1
Fertility	0.000263	APOB,ATP2B4,CHDH,GPX4,H2AFX,HSD17B4,HSPA4L,LAMB2,LCN2,MMP9

## Supplementary Materials

Supplementary materials can be found at http://www.mdpi.com/1422-0067/20/3/677/s1. Supplementary Table S1. Sperm concentration and motility in normozoospermic and asthenozoospermia testicular cancer patients, and normozoospermic infertile men without cancer (control group). Supplementary Table S2. List of primary and secondary antibodies.
