# Supplementary material for: Altered Molecular Pathways in the Proteome of Cryopreserved Sperm in Testicular Cancer Patients before Treatment
Source: Int J Mol Sci. 2019 Feb 5;20(3):677. doi: 10.3390/ijms20030677 (PMC6387327; doi:10.3390/ijms20030677)
Supplement: Supplementary file 1 [file ijms-20-00677-s001.zip › CCF-MDPI_PannerSelvam-Agarwal_1174-18-AND-1426469.pdf]

Cleveland Clinic
9500 Euclid Avenue Cleveland, Ohio 44195
(216) 444-5120 FAX (216) 445-6474
ARTphoto@ccf.org

January 22nd, 2019

Multidisciplinary Digital Publishing Institute (MDPI)
International Journal of Molecular Sciences
Dr. Maurizio Battino - Editor-in-Chief
Kaitlyn Wu - Assistant Editor
St. Alban-Anlage 66
4052 Basel, Switzerland
Tel: +41 61 683 77 34 Fax: +41 61 302 89 18
ISSN: 1422-0067 electronic; 1661-6596 print
Email: kaitlyn.wu@mdpi.com

On behalf of **Manesh Kumar Panner Selvam, PhD** (Research Fellow, Andrology, Glickman Urological and Kidney Institute), this letter serves as authorization by The Cleveland Clinic Foundation ("CCF"), which authorization includes that of **, Ashok Agarwal, PhD** and staff Illustrator **Terri Obrian, AA**, full time employee(s) of CCF to **Multidisciplinary Digital Publishing Institute**, to publish the work further described in Attachment A ("Artwork") solely for the purposes set forth herein. This authorization and license is an addendum to any agreement ("Author Agreement") signed between **CCF Staff** and **Multidisciplinary Digital Publishing Institute** related to the Article and supersedes any language related to the Artwork into the Article as described below, **Multidisciplinary Digital Publishing Institute** affirmatively agrees to this addendum and the licensing arrangement agreed to herein.

CCF grants to Publisher, a royalty-free, perpetual, non-exclusive license to use this Artwork as it appears in the work "**International Journal of Molecular Sciences**" article "**Altered molecular pathways in cryopreserved sperm proteome of testicular cancer patients before treatment**". Such use may be made of this Artwork in the Article in any format the Publisher utilizes. CCF further grants to Publisher the permission to use such Artwork in future publications and licensing arrangements as the Artwork appears within the Article.

All rights, title and interest in the Artwork shall remain the sole property of CCF, including but not limited to copyrights. The copyrights attribution may not be removed from this Artwork and the words, "Reprinted with permission, Cleveland Clinic Center for Medical Art & Photography ©2018-2019. All Rights Reserved." must accompany all such uses.

Sincerely,

Anthony Christovich, Coordinator II
Center for Medical Art and Photography-NA12
216-444-2592
christa@ccf.org

cc: J Loerch
T Obrian
A Agarwal
