# Supplementary material for: Altered Molecular Pathways in the Proteome of Cryopreserved Sperm in Testicular Cancer Patients before Treatment
Source: Int J Mol Sci. 2019 Feb 5;20(3):677. doi: 10.3390/ijms20030677 (PMC6387327; doi:10.3390/ijms20030677)
Supplement: Supplementary file 1 [file ijms-20-00677-s001.zip › Supplementary Table 1.docx]

**Supplementary Table 1:** Sperm concentration and motility in normozoospermic and asthenozoospermia testicular cancer patients, and normozoospermic infertile men (control group)

| **Parameter** | **Testicular cancer patients** | | **Control Group (n=20)** |
| --- | --- | --- | --- |
|  | **Normozoospermic (n=20)** | **Asthenozoospermic (n=20)** |  |
| Sperm concentration (10^6^/mL) | 23.48 ± 15.63 | 21.20 ± 15.37 | 29.20 ± 15.59 |
| **P value** | 0.1333 | 0.0620 |  |
| Sperm motility (%) | 64.25 ± 14.17 | 26.35 ± 8.87 | 59.30 ± 21.24 |
| **P value** | 0.3166 | < 0.0001 |  |

Statistical analysis was performed between the control group and normozoospermic and asthenozoospermic testicular cancer patients respectively. For all values, P < 0.05 indicate a significant difference based on the Mann-Whitney test. Values for sperm concentration and motility are presented as mean ± SD
