# Supplementary material for: Altered Molecular Pathways in the Proteome of Cryopreserved Sperm in Testicular Cancer Patients before Treatment
Source: Int J Mol Sci. 2019 Feb 5;20(3):677. doi: 10.3390/ijms20030677 (PMC6387327; doi:10.3390/ijms20030677)
Supplement: Supplementary file 1 [file ijms-20-00677-s001.zip › Supplementary Table 2.docx]

| **Primary** | | | | | **Secondary** | | | |
| --- | --- | --- | --- | --- | --- | --- | --- | --- |
| **Protein** | **Antibody** | **Source** | **Manufacturer** | **Dilution** | **Antibody** | **Source** | **Manufacturer** | **Dilution** |
| CCT3 | Anti-Human Rabbit IgG | Rabbit polyclonal | ab225878 | 1:2000 | Anti-Rabbit  Goat IgG | Goat polyclonal | ab97051 | 1:10000 |
| NDUFS1 |  | Rabbit monoclonal | ab157221 | 1:10000 |  |  |  |  |
| CD63 |  | Rabbit polyclonal | ab118307 | 1:500 |  |  |  |  |
| SERPINA5 | Anti-Human Mouse IgG | mouse polyclonal | ab172060 | 1:500 | Anti-Mouse  Rabbit IgG | Rabbit polyclonal | ab6728 | 1:10000 |

**Supplementary Table 2:** List of primary and secondary antibodies
